# Supplementary material for: Hot spot prediction in protein-protein interactions by an ensemble system
Source: BMC Syst Biol. 2018 Dec 31;12(Suppl 9):132. doi: 10.1186/s12918-018-0665-8 (PMC6311905; doi:10.1186/s12918-018-0665-8)
Supplement: Supplementary file 2 — All relevant MATLAB code. A simple Matlab implement of our predictor. (ZIP 19,742 kb) [file 12918_2018_665_MOESM2_ESM.zip › Additional file 2/Read ASA/ASA website/dbMPIKT/4YPGC.html]

 NetSurfP - Protein Surface Accessibility and Secondary Structure Predictions

  

|  |  |  |
| --- | --- | --- |
|  |  | NetSurfP - Protein Surface Accessibility and Secondary Structure Predictions  Technical University of Denmark |

  


---

```
# For publication of results, please cite:
# A generic method for assignment of reliability scores applied to solvent accessibility predictions.
# Bent Petersen, Thomas Nordahl Petersen, Pernille Andersen, Morten Nielsen and Claus Lundegaard
# BMC Structural Biology 2009, 9:51 doi:10.1186/1472-6807-9-51
#
# Column 1: Class assignment - B for buried or E for Exposed - Threshold: 25% exposure, but not based on RSA
# Column 2: Amino acid
# Column 3: Sequence name
# Column 4: Amino acid number
# Column 5: Relative Surface Accessibility - RSA
# Column 6: Absolute Surface Accessibility
# Column 7: Z-fit score for RSA prediction
# Column 8: Probability for Alpha-Helix
# Column 9: Probability for Beta-strand
# Column 10: Probability for Coil
E T  4YPGC                  1    0.748 103.761   0.145   0.003   0.003   0.994
E S  4YPGC                  2    0.600  70.273  -2.107   0.005   0.015   0.979
B C  4YPGC                  3    0.240  33.696  -0.703   0.018   0.019   0.964
E D  4YPGC                  4    0.526  75.854   0.382   0.058   0.017   0.925
B L  4YPGC                  5    0.100  18.402   1.090   0.246   0.004   0.750
E P  4YPGC                  6    0.372  52.801   1.208   0.321   0.003   0.675
E Q  4YPGC                  7    0.448  79.959  -0.725   0.694   0.003   0.303
E T  4YPGC                  8    0.380  52.637   0.280   0.622   0.015   0.363
B H  4YPGC                  9    0.174  31.614  -0.023   0.622   0.015   0.363
E S  4YPGC                 10    0.335  39.262  -0.430   0.622   0.015   0.363
E L  4YPGC                 11    0.430  78.770   0.080   0.717   0.014   0.269
B G  4YPGC                 12    0.244  19.211  -0.416   0.694   0.003   0.303
B S  4YPGC                 13    0.098  11.509  -0.824   0.782   0.003   0.216
E R  4YPGC                 14    0.364  83.241   0.756   0.923   0.002   0.076
E R  4YPGC                 15    0.419  96.020   1.296   0.970   0.001   0.030
B T  4YPGC                 16    0.027   3.745   0.128   0.970   0.001   0.030
B L  4YPGC                 17    0.082  14.941   0.636   0.970   0.001   0.030
E M  4YPGC                 18    0.505 101.111   1.883   0.970   0.001   0.030
B L  4YPGC                 19    0.112  20.452   1.219   0.970   0.001   0.030
B L  4YPGC                 20    0.041   7.525  -0.269   0.970   0.001   0.030
E A  4YPGC                 21    0.509  56.103   1.509   0.923   0.002   0.076
E Q  4YPGC                 22    0.562 100.337   1.218   0.858   0.002   0.139
B M  4YPGC                 23    0.068  13.547  -0.224   0.600   0.003   0.397
E R  4YPGC                 24    0.446 102.157  -0.336   0.246   0.004   0.750
E K  4YPGC                 25    0.659 135.536  -1.152   0.053   0.005   0.942
E I  4YPGC                 26    0.467  86.358  -1.539   0.016   0.005   0.979
E S  4YPGC                 27    0.305  35.793  -0.664   0.005   0.015   0.979
E L  4YPGC                 28    0.398  72.929  -1.264   0.018   0.019   0.964
E F  4YPGC                 29    0.452  90.636  -1.196   0.018   0.019   0.964
B S  4YPGC                 30    0.274  32.054  -0.704   0.053   0.005   0.942
B C  4YPGC                 31    0.100  14.082  -1.186   0.053   0.005   0.942
E L  4YPGC                 32    0.316  57.805  -0.767   0.257   0.016   0.727
E K  4YPGC                 33    0.620 127.616   0.076   0.181   0.016   0.803
E D  4YPGC                 34    0.311  44.772  -0.177   0.115   0.016   0.868
E R  4YPGC                 35    0.524 119.927  -0.272   0.053   0.043   0.903
E H  4YPGC                 36    0.483  87.858  -0.560   0.018   0.047   0.935
E D  4YPGC                 37    0.557  80.249  -0.143   0.018   0.088   0.893
B F  4YPGC                 38    0.052  10.436   0.393   0.018   0.047   0.935
E G  4YPGC                 39    0.497  39.138   0.606   0.018   0.047   0.935
B F  4YPGC                 40    0.117  23.422  -0.071   0.018   0.019   0.964
B P  4YPGC                 41    0.159  22.605  -0.750   0.246   0.004   0.750
E Q  4YPGC                 42    0.415  74.190   0.224   0.600   0.003   0.397
E E  4YPGC                 43    0.526  91.840   0.291   0.622   0.015   0.363
B E  4YPGC                 44    0.125  21.907  -1.212   0.522   0.016   0.462
B F  4YPGC                 45    0.222  44.515   0.271   0.339   0.016   0.645
E G  4YPGC                 46    0.547  43.080  -0.156   0.109   0.005   0.886
E N  4YPGC                 47    0.671  98.176  -0.530   0.115   0.016   0.868
E Q  4YPGC                 48    0.494  88.193  -0.360   0.257   0.016   0.727
B F  4YPGC                 49    0.231  46.462  -0.295   0.257   0.016   0.727
E Q  4YPGC                 50    0.498  88.961   0.825   0.622   0.015   0.363
E K  4YPGC                 51    0.389  79.935   0.684   0.970   0.001   0.030
E A  4YPGC                 52    0.297  32.707  -0.104   0.970   0.001   0.030
B E  4YPGC                 53    0.234  40.915  -0.130   0.970   0.001   0.030
B T  4YPGC                 54    0.070   9.764   0.144   0.988   0.000   0.012
B I  4YPGC                 55    0.063  11.655   0.782   0.988   0.000   0.012
E P  4YPGC                 56    0.205  29.033   1.744   0.988   0.000   0.012
B V  4YPGC                 57    0.032   4.918   0.612   0.988   0.000   0.012
B L  4YPGC                 58    0.021   3.863   1.403   0.988   0.000   0.012
B H  4YPGC                 59    0.100  18.190   1.068   0.988   0.000   0.012
B E  4YPGC                 60    0.196  34.294   0.950   0.988   0.000   0.012
B M  4YPGC                 61    0.029   5.763   0.724   0.988   0.000   0.012
B I  4YPGC                 62    0.034   6.327   0.266   0.988   0.000   0.012
E Q  4YPGC                 63    0.260  46.382   1.277   0.988   0.000   0.012
B Q  4YPGC                 64    0.172  30.719   0.652   0.988   0.000   0.012
B I  4YPGC                 65    0.026   4.755   0.858   0.988   0.000   0.012
B F  4YPGC                 66    0.089  17.923   0.279   0.988   0.000   0.012
E N  4YPGC                 67    0.330  48.312   1.050   0.970   0.001   0.030
B L  4YPGC                 68    0.060  11.059   0.607   0.970   0.001   0.030
B F  4YPGC                 69    0.053  10.617  -0.082   0.923   0.002   0.076
E S  4YPGC                 70    0.329  38.500  -0.270   0.600   0.003   0.397
E T  4YPGC                 71    0.448  62.151   0.255   0.246   0.004   0.750
E K  4YPGC                 72    0.526 108.260  -1.361   0.109   0.005   0.886
E D  4YPGC                 73    0.468  67.396  -1.938   0.016   0.005   0.979
E S  4YPGC                 74    0.428  50.103  -1.496   0.016   0.005   0.979
E S  4YPGC                 75    0.384  45.052  -1.025   0.053   0.005   0.942
E A  4YPGC                 76    0.491  54.141  -1.212   0.246   0.004   0.750
E A  4YPGC                 77    0.471  51.948   0.081   0.321   0.003   0.675
B W  4YPGC                 78    0.161  38.648   0.512   0.522   0.016   0.462
E D  4YPGC                 79    0.380  54.744   0.746   0.522   0.016   0.462
E E  4YPGC                 80    0.577 100.715   1.119   0.923   0.002   0.076
E T  4YPGC                 81    0.548  76.063   1.559   0.970   0.001   0.030
B L  4YPGC                 82    0.191  35.027   1.590   0.970   0.001   0.030
B L  4YPGC                 83    0.096  17.486   0.787   0.988   0.000   0.012
E D  4YPGC                 84    0.435  62.655   0.941   0.988   0.000   0.012
E K  4YPGC                 85    0.511 105.133   1.775   0.988   0.000   0.012
B F  4YPGC                 86    0.036   7.145   0.179   0.988   0.000   0.012
B Y  4YPGC                 87    0.135  28.828   1.043   0.970   0.001   0.030
E T  4YPGC                 88    0.432  59.974  -0.183   0.970   0.001   0.030
B E  4YPGC                 89    0.241  42.190   0.917   0.970   0.001   0.030
B L  4YPGC                 90    0.031   5.658   0.415   0.970   0.001   0.030
B Y  4YPGC                 91    0.252  53.810   0.578   0.970   0.001   0.030
E Q  4YPGC                 92    0.401  71.672   0.781   0.970   0.001   0.030
B Q  4YPGC                 93    0.111  19.753  -0.206   0.970   0.001   0.030
B L  4YPGC                 94    0.050   9.118   0.417   0.970   0.001   0.030
E N  4YPGC                 95    0.527  77.167   1.045   0.970   0.001   0.030
E D  4YPGC                 96    0.331  47.639   1.000   0.923   0.002   0.076
B L  4YPGC                 97    0.045   8.313  -0.444   0.923   0.002   0.076
E E  4YPGC                 98    0.487  85.149   0.887   0.923   0.002   0.076
E A  4YPGC                 99    0.504  55.497   1.360   0.858   0.002   0.139
B C  4YPGC                100    0.068   9.519   0.250   0.858   0.002   0.139
B V  4YPGC                101    0.164  25.176   0.316   0.802   0.014   0.185
E I  4YPGC                102    0.457  84.489  -0.245   0.717   0.014   0.269
E Q  4YPGC                103    0.586 104.695   0.325   0.622   0.015   0.363
E G  4YPGC                104    0.343  26.955  -0.949   0.622   0.015   0.363
E V  4YPGC                105    0.356  54.717  -1.000   0.660   0.049   0.291
E G  4YPGC                106    0.540  42.498  -0.536   0.605   0.105   0.290
E V  4YPGC                107    0.501  76.973  -0.521   0.502   0.102   0.396
E T  4YPGC                108    0.662  91.806  -0.765   0.278   0.093   0.628
E E  4YPGC                109    0.579 101.099  -1.117   0.191   0.086   0.723
E T  4YPGC                110    0.459  63.705  -1.472   0.113   0.043   0.844
E P  4YPGC                111    0.353  50.105  -1.430   0.053   0.043   0.903
E L  4YPGC                112    0.332  60.826  -1.318   0.053   0.043   0.903
E M  4YPGC                113    0.381  76.238  -0.738   0.053   0.043   0.903
E K  4YPGC                114    0.600 123.399  -1.096   0.113   0.043   0.844
E E  4YPGC                115    0.567  99.055  -1.283   0.184   0.043   0.773
E D  4YPGC                116    0.509  73.419  -1.480   0.181   0.016   0.803
B S  4YPGC                117    0.261  30.589  -0.959   0.522   0.016   0.462
E I  4YPGC                118    0.384  70.966   0.341   0.802   0.014   0.185
E L  4YPGC                119    0.377  69.102   0.055   0.879   0.010   0.111
E A  4YPGC                120    0.307  33.831   0.983   0.975   0.003   0.022
B V  4YPGC                121    0.038   5.779  -0.332   0.970   0.001   0.030
E R  4YPGC                122    0.331  75.822   1.199   0.970   0.001   0.030
E K  4YPGC                123    0.533 109.638   1.530   0.970   0.001   0.030
B Y  4YPGC                124    0.122  26.071   1.018   0.970   0.001   0.030
B F  4YPGC                125    0.049   9.754  -0.239   0.970   0.001   0.030
E Q  4YPGC                126    0.430  76.887   1.338   0.970   0.001   0.030
E R  4YPGC                127    0.313  71.746   1.401   0.970   0.001   0.030
B I  4YPGC                128    0.025   4.699   0.829   0.970   0.001   0.030
B T  4YPGC                129    0.247  34.231   0.701   0.970   0.001   0.030
E L  4YPGC                130    0.495  90.561   1.943   0.970   0.001   0.030
B Y  4YPGC                131    0.133  28.443   1.480   0.970   0.001   0.030
B L  4YPGC                132    0.047   8.569  -0.010   0.923   0.002   0.076
E K  4YPGC                133    0.527 108.424   1.231   0.858   0.002   0.139
E E  4YPGC                134    0.742 129.680   0.953   0.502   0.002   0.495
E K  4YPGC                135    0.447  91.907   0.650   0.109   0.005   0.886
E K  4YPGC                136    0.682 140.267   0.118   0.016   0.005   0.979
B Y  4YPGC                137    0.207  44.172   0.082   0.016   0.005   0.979
B S  4YPGC                138    0.192  22.526  -0.366   0.176   0.004   0.820
E P  4YPGC                139    0.413  58.605  -0.266   0.600   0.003   0.397
B C  4YPGC                140    0.171  23.980   0.314   0.802   0.014   0.185
B A  4YPGC                141    0.030   3.339   0.386   0.938   0.007   0.055
B W  4YPGC                142    0.094  22.583   0.636   0.975   0.003   0.022
B E  4YPGC                143    0.253  44.217   0.827   0.975   0.003   0.022
B V  4YPGC                144    0.157  24.116   1.555   0.975   0.003   0.022
B V  4YPGC                145    0.021   3.258   1.383   0.975   0.003   0.022
B R  4YPGC                146    0.160  36.754   1.330   0.988   0.000   0.012
E A  4YPGC                147    0.231  25.445   0.743   0.988   0.000   0.012
B E  4YPGC                148    0.136  23.672   1.051   0.988   0.000   0.012
B I  4YPGC                149    0.026   4.828   0.372   0.988   0.000   0.012
E M  4YPGC                150    0.283  56.728   0.896   0.988   0.000   0.012
E R  4YPGC                151    0.339  77.539   0.642   0.970   0.001   0.030
B S  4YPGC                152    0.059   6.891   0.398   0.970   0.001   0.030
B F  4YPGC                153    0.059  11.841   0.605   0.970   0.001   0.030
E S  4YPGC                154    0.317  37.188   0.207   0.970   0.001   0.030
B L  4YPGC                155    0.217  39.733   0.526   0.970   0.001   0.030
B S  4YPGC                156    0.078   9.130  -0.432   0.923   0.002   0.076
E T  4YPGC                157    0.348  48.226  -0.063   0.858   0.002   0.139
E N  4YPGC                158    0.494  72.336   1.242   0.694   0.003   0.303
B L  4YPGC                159    0.211  38.726  -0.281   0.502   0.002   0.495
E Q  4YPGC                160    0.516  92.211   0.141   0.246   0.004   0.750
E E  4YPGC                161    0.908 158.645   1.295   0.003   0.003   0.994

Explain the output.  Go back.
```

---
